# Effect of salivary pH variations on the adhesive performance of Transbond XT and Orthocem in orthodontic bracket bonding

**DOI:** 10.2340/biid.v13.45568

**Published:** 2026-03-23

**Authors:** Lismory Karina Leiton Narváez, Marjory Elizabeth Vaca Zapata, Mauricio Aguirre Balseca, Karina Maria Salvatore Freitas, Yalil Rodríguez

**Affiliations:** a Department of Orthodontics, University of the Hemispheres, Quito, Ecuador; b Department of Endodontics, University of the Hemispheres, Quito, Ecuador; c Department of Orthodontics, Ingá University Center Uningá, Maringá, Brazil

**Keywords:** Orthodontic brackets, shear bond strength, Transbond XT, Orthocem, salivary pH, adhesive systems

## Abstract

**Objective:**

This in vitro study aimed to evaluate the shear bond strength of orthodontic brackets bonded to acrylic resin specimens with two adhesive systems, Transbond XT and Orthocem, under different salivary pH levels (4, 5, 6, and 7).

**Materials and methods:**

A total of 120 acrylic resin specimens (Brava Block^®^, FGM) were prepared and randomly assigned to three groups: control bonded with Transbond XT and Orthocem with no salivary exposure (*n* = 24), Transbond XT (*n* = 48), and Orthocem (*n* = 48). Each adhesive group was subdivided and immersed in artificial saliva at pH 4, 5, 6, or 7 for 30 days. Metal premolar brackets (Vector+ Roth prescription, Aditek^®^) were bonded following manufacturers’ protocols and subjected to shear bond strength testing using a Universal Testing Machine, following ISO 4049 standards. Data were analyzed with Analysis of Variance (ANOVA), *t*-test, and Tukey tests at a significance level of *p* < 0.05.

**Results:**

Transbond XT consistently showed higher bond strength than Orthocem across all pH levels. Maximum resistance was observed at pH 7 (21.19 ± 5.06 MPa), followed by the control group (19.64 ± 5.28 MPa). Orthocem demonstrated significantly lower values, ranging from 9.51 ± 3.87 MPa (pH 5) to 4.94 ± 2.15 MPa (pH 6). Acidic conditions reduced the bond strength of both adhesives, but Transbond XT maintained clinically reliable values, while Orthocem was more susceptible to performance decline at moderately acidic levels.

**Conclusions:**

Salivary pH directly influenced adhesive performance. Transbond XT demonstrated superior resistance under all conditions, whereas Orthocem showed lower but still clinically acceptable shear bond strength values, except at pH 6, where a marked decrease was observed.

## Introduction

The increasing demand for orthodontic treatment across all age groups has emphasized the importance of achieving both functional occlusion and esthetic outcomes. A key determinant of treatment success is the secure bonding of brackets, as bracket debonding remains one of the most common complications during therapy, directly affecting treatment efficiency and patient satisfaction [1, 2]. Early orthodontic techniques relied on banding, which was progressively replaced by direct bonding systems to improve esthetics, hygiene, and chairside efficiency [3, 4].

Despite advancements in adhesive technology, bracket failure remains a frequent problem, with reported debonding rates ranging from 0.5 to 28%, resulting in prolonged treatment, additional costs, enamel wear, and potential dentin hypersensitivity [2, 5]. Transbond XT (3M Unitek) has long been considered the gold standard orthodontic adhesive due to its excellent shear bond strength and clinical reliability [6, 7]. However, its high cost represents a limitation in many regions. Orthocem (FGM Dental Group), a more economical alternative, has shown acceptable clinical performance but lower bond strength compared to Transbond XT [8].

Adhesive bond strength is influenced not only by the adhesive system itself but also by intraoral environmental conditions. The oral cavity represents a dynamic environment, with fluctuations in pH, temperature, salivary composition, and mechanical loading, all of which may compromise adhesive performance [9, 10]. Among these factors, salivary pH is particularly critical. Acidic conditions (pH ≤ 5.5), often induced by dietary acids, bacterial metabolism, or inadequate oral hygiene, can promote enamel demineralization and reduce the mechanical retention required for bracket adhesion [11, 12]. Experimental studies have confirmed that shear bond strength is significantly reduced under acidic pH, increasing the likelihood of bracket failure [11, 13]. Conversely, neutral or slightly basic conditions provide more favorable environments for adhesive stability.

The effect of saliva and pH variations on bonding is also clinically relevant due to the difficulty of achieving perfect enamel isolation in orthodontic practice. Even brief exposure of the etched surface to saliva can reduce bond strength by up to 50% through the formation of an organic layer that hinders resin infiltration [14, 15]. Additionally, corrosion and structural changes in brackets and bonding interfaces have been documented under acidic conditions, further compromising treatment durability [16, 17].

Although Transbond XT has been extensively studied, evidence regarding the performance of Orthocem under different salivary pH levels remains limited. Given its growing clinical use due to affordability, further studies are necessary to validate its reliability compared to the benchmark adhesive. Understanding how salivary pH influences bond strength is essential for guiding adhesive selection, improving clinical outcomes, and minimizing bracket failure.

Therefore, the objective of this in vitro study was to evaluate the shear bond strength of brackets bonded to acrylic resin specimens with two adhesive systems, Transbond XT and Orthocem, under different salivary pH levels of 4, 5, 6, and 7.

## Materials and methods

### Sample size calculation

An a priori power analysis was performed using G*Power software (version 3.1, Heinrich Heine University, Düsseldorf, Germany) based on a one-way ANOVA design with five pH conditions within each adhesive system, using shear bond strength (MPa) as the primary outcome. The calculation assumed a large effect size (Cohen’s *f* = 0.40) between pH levels, a significance level of α = 0.05, and a statistical power of 80%. Under these parameters, a minimum of 10 specimens per subgroup was required. To compensate for potential specimen loss or experimental errors, the sample size was increased to 12 specimens per subgroup, resulting in a total of 120 acrylic resin specimens distributed across the experimental conditions.

### Study design

This experimental in vitro study evaluated the shear bond strength of orthodontic brackets bonded with two adhesive systems (Transbond XT and Orthocem) under different salivary pH conditions. A total of 120 acrylic resin specimens were prepared from Brava Block^®^ resin blocks (FGM Dental Group, Brazil), standardized to dimensions of 14 × 14 × 3 mm.

### Specimen preparation

Resin blocks were sectioned into standardized specimens using a diamond disk (1.772 in × 0.012 in) mounted on a high-speed cutting machine (25,000 rpm) with continuous water irrigation. The surfaces were polished sequentially with silicon carbide abrasive papers (600, 1000, and 1200 grit) and stored in sterile plastic containers until use. Each specimen was then etched with hydrofluoric acid, rinsed, and dried. A thin layer of the respective adhesive system used for each group was applied, followed by light-curing in four directions (mesial, distal, incisal, and cervical) using a Woodpecker O-Light LED curing unit [11].

### Experimental groups

Specimens were randomly assigned into three experimental groups:

- Group 1 – Control (*n* = 24): Brackets were bonded using either Transbond XT (*n* = 12) or Orthocem (*n* = 12). No exposure to artificial saliva was performed.
- Group 2 – Transbond XT (*n* = 48): Specimens were divided into four subgroups (*n* = 12 each) and immersed in artificial saliva at pH 7, 6, 5, and 4, respectively.
- Group 3 – Orthocem (*n* = 48): Specimens were similarly divided into four subgroups (*n* = 12 each) and immersed in artificial saliva at pH 7, 6, 5, and 4, respectively.

Both adhesives complied with American Dental Association (ADA) Specification No. 27 and No. 66 [18, 19].

### Bracket bonding procedure

Conventional stainless steel premolar brackets (Vector+ Roth prescription 0.022 × 0.028-in slot; Aditek, Brazil) were used. In Group 1 (control), brackets were bonded with either:

- Transbond XT: primer + adhesive, followed by light-curing for 5 seconds.
- Orthocem: Ambar Advanced Polymerization System (APS) primer + Orthocem, followed by light-curing for 10 seconds.

For Groups 2 and 3, the same bonding protocol was followed according to the adhesive system, and specimens were subsequently immersed in artificial saliva with different pH levels for 30 days.

### Artificial saliva preparation

Artificial saliva (Saliv^®^, Lamosan Laboratories, Peru) was used to simulate the oral environment. Its composition included electrolytes (Na⁺ 2–21 mmol/L, K⁺ 10–36 mmol/L, Ca²⁺ 1.2–2.8 mmol/L, Mg²⁺ 0.08–0.5 mmol/L, Cl⁻ 5–40 mmol/L, HCO₃⁻ ~25 mmol/L, PO₄³⁻ 1.4–39 mmol/L), xylitol, and preservatives (nipagin–nipasol complex) [20].

### Immersion protocol

- Group 1 (Control, Transbond XT and Orthocem): No immersion in artificial saliva. The specimens were stored for 30 days in a sterile metal container.
- Group 2 (Transbond XT): Specimens immersed in artificial saliva at pH 4, 5, 6, or 7 (*n* = 12 per subgroup).
- Group 3 (Orthocem): Specimens immersed in artificial saliva at pH 4, 5, 6, or 7 (*n* = 12 per subgroup).

All specimens of groups 2 and 3 were immersed in artificial saliva for 30 days, with pH levels adjusted and monitored daily.

### Shear bond strength test

After immersion, specimens were subjected to shear bond strength testing using a Universal Testing Machine (MTS T5002, 110/120 V, 60 Hz, 300 kg load capacity, 20 in/min crosshead speed). Brackets were positioned vertically, and a chisel-edge plunger was applied at the bracket–resin interface until bond failure occurred. Shear bond strength was calculated by using the load at failure provided directly by the Universal Testing Machine, recorded in Newtons (N). This value was divided by the bracket base area to obtain the final shear bond strength in megapascals (MPa). All brackets used in the experiment had the same base area, ensuring consistency across all experimental groups. The testing procedure followed ISO 4049 standards [21, 22].

### Statistical analysis

Data were analyzed using SPSS (version 23, IBM, Spanish version). Normality and homogeneity of variance were verified using Shapiro–Wilk, Levene, and Kolmogorov–Smirnov tests. Comparative analyses included:

- One-way ANOVA to compare bond strength across groups and pH levels.
- Independent *t*-tests to compare the two adhesive systems
- Tukey’s post-hoc test for multiple comparisons.

The significance level was set at *p* < 0.05.

## Results

**Figure 1:**
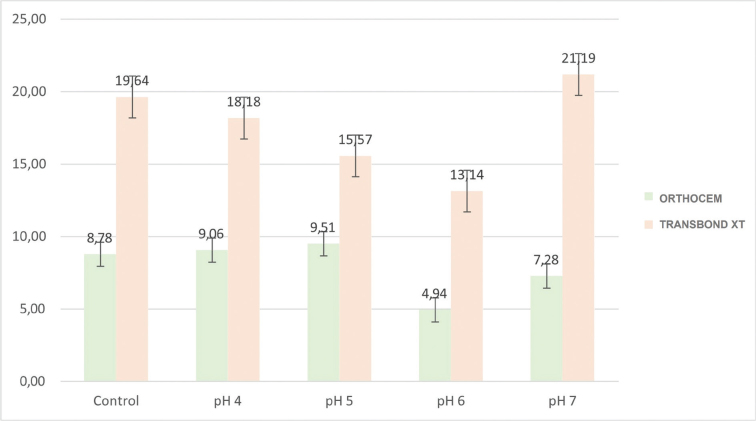
Shear bond strength of brackets under different salivary pH levels using two adhesive systems.

The shear bond strength results are presented in Figure 1. The ANOVA found shear bond strength values to vary between groups (*P* < 0.001). The shear bond strength values were significantly higher for Transbond XT than Orthocem at all five storage conditions (Table 1).

**Table 1:** Student’s *t*-test comparing the shear bond strength of brackets between the Transbond XT and Orthocem (FGM) adhesive systems.

	ORTHOCEM		TRANSBOND		95% CI			*p-value*
	Mean	SD	Mean	SD	Mean Diff	Min.	Máx.	
**Control**	8.78	2.69	19.64	5.28	-10.86	-9.21	-12.51	*0.000*
**pH 4**	9.06	2.06	18.18	6.84	-9.12	-6.08	-12.16	*0.000*
**pH 5**	9.51	3.87	15.57	4.34	-6.06	-5.77	-6.36	*0.001*
**pH 6**	4.94	2.15	13.14	5.04	-8.20	-6.36	-10.04	*0.000*
**pH 7**	7.28	2.01	21.19	5.06	-13.92	-11.98	-15.85	0.000

For Transbond XT, storage at pH 6 resulted in significantly lower bond strength than the control and storage at pH 7, while storage at pH 5 also gave a significantly lower bond strength than did storage at pH 7 (Table 2).

**Table 2:** ANOVA–Tukey test of shear bond strength of brackets under different salivary pH levels using the Transbond XT adhesive system.

pH		Mean Diff	CI 95%		*p-value*
			Min.	Max.	
**Control**	**pH4**	1.46	-4.73	7.65	*0.96*
	**pH5**	4.07	-2.12	10.26	*0.35*
	**pH6**	6.49*	0.31	12.69	*0.04*
	**pH7**	-1.55	-7.74	4.64	*0.95*
**pH4**	**pH5**	2.61	-3.58	8.80	*0.76*
	**pH6**	5.04	-1.15	11.23	*0.16*
	**pH7**	-3.01	-9.20	3.18	*0.65*
**pH5**	**pH6**	2.43	-3.76	8.62	*0.80*
	**pH7**	-5.62	-11.81	0.57	*0.09*
**pH6**	**pH7**	-8.04*	-14.24	-1.86	0.00

For Orthocem, storage at pH 6 resulted in significantly lower bond strength than did the control and storage at pH 4 and pH 5 (Table 3).

**Table 3:** ANOVA–Tukey test of shear bond strength of brackets under different salivary pH levels using the Orthocem (FGM) adhesive system.

pH		Mean Diff	CI 95%		*p-value*
			Min.	Max.	
**Control**	**pH4**	-0.28	-3.33	2.77	1.00
	**pH5**	-0.73	-3.78	2.32	0.96
	**pH6**	3.83*	0.79	6.89	0.01
	**pH7**	1.50	-1.54	4.55	0.64
**pH4**	**pH5**	-0.45	-3.49	2.60	0.99
	**pH6**	4.11*	1.07	7.17	0.00
	**pH7**	1.79	-1.26	4.83	0.47
**pH5**	**pH6**	4.56*	1.52	7.61	0.00
	**pH7**	2.23	-0.82	5.28	0.25
**pH6**	**pH7**	-2.33	-5.38	0.72	0.21

## Discussion

The aim of this study was to evaluate the shear bond strength of orthodontic brackets cemented with two adhesive systems, Transbond XT and Orthocem, under different salivary pH levels (4, 5, 6, and 7). Our findings revealed significant differences between the materials, consistently favoring Transbond XT, which demonstrated higher bond strength across all conditions.

This study provides relevant insights for orthodontic patients, as salivary pH fluctuations can significantly influence adhesive properties and bracket retention. The two adhesive systems evaluated, Transbond XT and Orthocem (FGM), were selected for their widespread clinical use and potential to provide reliable bond strength when applied according to manufacturers’ protocols.

Acrylic resin blocks were deliberately selected as the bonding substrate in this study to ensure a high level of standardization and reproducibility across specimens. The use of extracted human teeth introduces inherent variability related to enamel thickness, mineralization degree, fluoride exposure, age, and previous chemical or mechanical alterations, which may significantly influence bond strength outcomes. By using resin blocks with uniform composition and surface characteristics, substrate-related confounding factors were minimized, allowing the isolated evaluation of the effect of salivary pH on the adhesive performance of the two bonding materials.

In addition, bonding to resin-based substrates has direct clinical relevance, particularly in adult orthodontic patients who frequently present with provisional crowns, composite restorations, repaired surfaces, or indirect restorations. Under these conditions, orthodontic brackets are often bonded to non-enamel surfaces, and adhesive behavior may differ substantially from that observed on natural enamel. Therefore, acrylic resin blocks provide a controlled and clinically meaningful model for assessing adhesive performance under challenging environmental conditions.

Previous studies have demonstrated that orthodontic adhesives behave differently on restorative materials. Hellak et al. [23] noted significant variations in shear bond strength when adhesives were applied to composite resin and ceramic surfaces. Similarly, Tallani et al. [24] and Uslu et al. [25] reported that the interaction between adhesives and restorative materials can affect both initial retention and long-term stability. In this context, acrylic resin blocks offer a reproducible substrate that simulates bonding on provisional or restorative materials.

The performance of the adhesives under different pH conditions was particularly noteworthy. Transbond XT showed optimal behavior under neutral or near-neutral conditions (control and pH 7), with values (19.64–21.19 MPa) higher than those commonly reported in the literature [23–25]. Although these values ensure excellent bracket stability, excessively high bond strength may complicate debonding and increase the risk of enamel damage or residual adhesive [26]. This highlights the need to balance adequate retention with safe debonding.

The effect of salivary pH was particularly evident. As expected, Transbond XT performed best in neutral or near-neutral conditions (control and pH 7), while acidic conditions significantly reduced bond strength. This pattern aligns with previous studies showing that lower pH weakens the adhesive interface by compromising the polymeric matrix and altering enamel microstructure [10, 27]. Nevertheless, even at acidic pH, Transbond XT maintained values above the clinically acceptable threshold of 5.9–7.8 MPa suggested by Ganiger et al. [13], reinforcing its predictability in challenging intraoral conditions.

In contrast, Orthocem exhibited significantly lower bond strength, with values ranging between 4.94 and 9.51 MPa depending on the pH level. Although these values were consistently lower than those of Transbond XT, most of them remained above the minimum clinically acceptable value of 5.9 MPa [13, 26]. Notably, Orthocem demonstrated a dramatic reduction at pH 6 (4.94 MPa), highlighting its susceptibility to moderate acidity. Similar trends have been described by Contreras et al. [11] and Ganiger et al. [13], who reported that Orthocem’s performance is more sensitive to operator technique, humidity, and unfavorable pH variations. This suggests that Orthocem may be a viable, cost-effective alternative in stable environments but requires caution in patients with acidic salivary profiles or poor oral hygiene.

Although low pH is generally expected to reduce adhesive performance, several studies show that the effect of acidity on resin-based orthodontic adhesives is not linear. The greatest degradation typically occurs at moderately acidic conditions (pH 5–6), not necessarily at extremely acidic pH. Iosif et al. [10] demonstrated that resin cements undergo more hydrolytic breakdown around pH 5–6 due to increased water sorption and polymer network disruption, while very low pH may reduce water uptake and produce less hydrolysis. Similar behavior was reported by Contreras et al. [11] and Ganiger et al. [28], who found that Orthocem and other adhesives show their largest reduction in bond strength at intermediate acidic pH levels. In addition, because our specimens were bonded to acrylic resin rather than enamel, no substrate demineralization occurred at pH 4, minimizing the effect of extreme acidity. Artificial saliva also contains buffering ions that partially neutralize very low pH solutions. Together, these factors help explain why storage at pH 4 did not reduce bond strength as much as storage at pH 5 or pH 6 in the present study.

The clinical implications of these findings are relevant. Bracket failure remains a common complication in orthodontics, with incidence ranging between 0.5 and 17.6% during treatment [2]. Failures increase chair time, extend treatment duration, and can cause unnecessary enamel wear during rebonding. Adhesive systems that are less sensitive to environmental factors, such as Transbond XT, provide greater predictability and reduce the risk of bond failure under variable salivary conditions. On the other hand, Orthocem’s lower cost and acceptable bond strength make it a practical option in resource-limited settings, provided that pH fluctuations are considered and preventive measures, such as dietary counseling and the use of neutralizing agents, are implemented.

Our results corroborate with prior literature showing that acidic salivary conditions are detrimental to bond performance [11, 12]. The findings also highlight the importance of patient-specific factors, such as diet, salivary buffering capacity, and oral hygiene, in determining adhesive success. These variables should be carefully assessed when selecting an adhesive system, especially in high-risk patients.

A strength of this study is the standardized in vitro design using acrylic resin blocks, which minimized substrate variability. Despite this advantage, the use of acrylic resin instead of human enamel represents a limitation of the present study. Resin substrates do not replicate the complex microstructure, chemical composition, and biological behavior of enamel, particularly with respect to acid-induced demineralization and resin–enamel hybrid layer formation. As a result, the absolute shear bond strength values observed in this study cannot be directly extrapolated to clinical bonding on natural enamel.

Furthermore, because acrylic resin does not undergo mineral loss under acidic conditions, the influence of low pH on enamel integrity and its interaction with adhesive systems could not be assessed. Therefore, the findings of this in vitro study should be interpreted as a comparative assessment of adhesive performance under controlled conditions rather than a direct simulation of clinical enamel bonding. Future studies using extracted teeth, thermocycling, and long-term aging protocols are recommended to complement these findings and enhance clinical extrapolation.

In summary, the results confirm that Transbond XT maintains superior shear bond strength compared to Orthocem across different salivary pH levels. Orthocem demonstrated lower but clinically acceptable performance, although its susceptibility to moderately acidic conditions warrants caution. Clinicians should consider salivary pH as a potential risk factor in adhesive selection to ensure treatment stability and minimize bracket failure.

## Conclusions

This in vitro study showed that salivary pH directly affects bracket adhesion. Transbond XT achieved higher shear bond strength at all pH levels, confirming greater stability, while Orthocem presented lower but clinically acceptable values, except for a marked decrease at pH 6. These results highlight the need to consider salivary pH variations in clinical practice when selecting the adhesive system to ensure treatment stability.
